# Intranasal neuropeptide Y suppresses food intake, reduces sucrose preference and increases circulating corticosterone in obese mice

**DOI:** 10.1016/j.neurot.2026.e01100

**Published:** 2026-10-22

**Authors:** Maike Haag, Karolina Herceg, Chi Kin Ip, Angela Jačan, Florian Reichmann, Herbert Herzog, Andreas Meinitzer, Peter Holzer, Ahmed M. Hassan, Aitak Farzi

**Affiliations:** a Otto Loewi Research Center, Division of Pharmacology, Medical University of Graz, Graz, Austria; b Department of Internal Medicine, Division of Angiology, Medical University of Graz, Graz, Austria; c School of Clinical Medicine, St. Vincent's Clinical School, UNSW Medicine & Health, UNSW Sydney, Sydney, NSW, 2010, Australia; d CBmed GmbH-Center for Biomarker Research in Medicine, Graz, Austria; e Eating Disorder Research Program, St. Vincent's Centre for Applied Medical Research, 405 Liverpool Street, Darlinghurst, Sydney, NSW, 2010, Australia; f Clinical Institute of Medical and Chemical Laboratory Diagnostics, Medical University of Graz, Graz, Austria

**Keywords:** Anorexia, High-fat diet, Reward sensitivity, Obesity, Intranasal delivery, Peptides

## Abstract

Obesity is associated with an increased risk of psychiatric disorders and altered reward processing. Neuropeptide Y (NPY) links metabolic state, reward-driven behavior, and emotional processing. We therefore tested whether intranasal (i.n.) NPY modifies high-fat diet (HFD)-induced behavioral alterations. Male C57BL/6J mice were fed HFD (48% kcal fat) or control diet for 8 weeks and assessed for anxiety-like behavior using the light/dark box test (LDT), self-care behavior using the splash test, and sucrose-directed ingestive behavior using sucrose intake and sucrose preference (SP). A separate cohort of control- and HFD-fed mice received i.n. NPY or vehicle, followed by assessment of SP and ingestive behavior after 3, 24 and 48 h. Neuroendocrine and inflammatory parameters were measured in blood, brain, and colon. HFD reduced sucrose-directed ingestive behavior, reflected by reduced sucrose intake and SP, without significantly affecting anxiety-like behavior in the LDT or self-care behavior in the splash test. HFD reduced hypothalamic *Npy* mRNA and peptide levels as well as circulating corticosterone, while the evaluated inflammation-related markers remained unchanged. I.n. NPY had no effect after 24 or 48 h but reduced sucrose intake and SP, and increased circulating corticosterone 3 h after administration specifically in HFD-fed animals. In contrast, i.n. NPY reduced food intake and body weight after 3 h, independent of diet. Because these effects were consistent with neuropeptide Y receptor Y2 (NPY2R)-mediated actions, we tested i.n. NPY in HFD-fed *Npy2r*-deficient mice. In these mice, i.n. NPY did not affect sucrose intake, SP, food intake, or corticosterone, consistent with a potential involvement of NPY2R.

## Introduction

Obesity is a risk factor for the development of anxiety and depressive disorders in humans [1], [2]. In line with clinical evidence, animal models of diet-induced obesity (DIO) frequently display anxiety-like and depression-like behavior, as well as blunted responses to rewarding stimuli [3], [4], [5], [6]. This relationship between metabolic status and mental health is not surprising, given that appetite, mood regulation, and reward processing rely on overlapping neural and molecular mechanisms [7], [8].

Among these overlapping mechanisms, the neuropeptide Y (NPY) system plays a central role. Hypothalamic NPY is a key regulator of appetite and energy balance. NPY promotes feeding primarily via neuropeptide Y receptor Y1 (NPY1R) and neuropeptide Y receptor Y5 (NPY5R), while neuropeptide Y receptor Y2 (NPY2R) often functions as a presynaptic inhibitory autoreceptor that limits NPY release; therefore, selective activation of NPY2R in the hypothalamus or peripherally has an anorexigenic effect [9], [10]. Furthermore, NPY regulates the rewarding effects of palatable foods. This effect is mediated, at least in part, by projections from hypothalamic NPY neurons (particularly in the arcuate nucleus) to the ventral tegmental area and the nucleus accumbens [11]. Beyond its role in the regulation of feeding behavior, NPY enhances stress resilience and regulates affective states [12]. Through its effects on the hypothalamic-pituitary-adrenal (HPA) axis, NPY contributes to the coordination of stress responses while also influencing blood glucose homeostasis and appetite. In addition, NPY modulates inflammatory processes that are increasingly recognized as contributors to neuropsychiatric disorders [13], [14]. One well-characterized mechanism linking inflammation to altered brain function is the tryptophan-kynurenine pathway. Inflammatory mediators stimulate the enzyme indoleamine 2,3-dioxygenase (IDO), which diverts tryptophan metabolism away from serotonin synthesis toward the production of kynurenine and its neuroactive metabolites, which promote depressive- and anxiety-like behaviors [15].

DIO in experimental animals reduces hypothalamic and hippocampal NPY expression [5], [16]. While this downregulation in the hypothalamus may serve as an adaptive mechanism to limit food intake and protect against morbid obesity [17], this benefit may come at the expense of NPY's role in reward processing and stress resilience. Reduced NPY expression has been observed in several brain regions in animal models of depression exhibiting anhedonia, as well as in postmortem brain tissue from patients with major depressive disorder [18], [19], [20]. Furthermore, administration of NPY either intracerebrally or intranasally (i.n.) reduces depression-like behaviors in animal models [21], [22]. Therefore, reduced NPY signaling observed in animal models of DIO may contribute to the behavioral alterations seen in these animals, but to the best of our knowledge, this mechanism has not yet been systematically investigated.

In this work, we assessed the behavioral changes induced by a moderate high-fat diet (HFD; 48% of calories from fat), a composition that closely reflects human Western diet, in C57BL/6J mice [23]. In parallel, we examined the effects of this dietary intervention on plasma corticosterone levels, inflammatory markers, and the tryptophan-kynurenine pathway. To explore the contribution of reduced NPY signaling to these behavioral and neuroendocrine alterations, we further investigated whether i.n. administration of NPY could reverse the observed changes. Since the effects of i.n. NPY in our work appeared to align with NPY2R-mediated actions, we further tested the effect of intranasal NPY in *Npy2r*-knockout (KO) mice.

## Material and methods

### Ethics statement

All experiments were approved by an ethical committee at the Federal Ministry of Science, Research and Economy of the Republic of Austria (permit BMWFW-66.010/0131-WF/II/3b/2014, permit BMWFW-66.010/0050-WF/V/3b/2017, permit BMBWF-66.010/0146-V/3b/2018, and permit 2021-0.785.840). All procedures were conducted according to the Directive of the European Parliament and of the Council of September 22, 2010 (2010/63/EU).

### Experimental animals

The experimental protocols 1–3 (see below) in this study were conducted using male C57BL/6J mice (Charles River, Sulzfeld, Germany) at 8 weeks of age. The animals were housed two or three per cage with a temperature set at 21 °C and humidity at 50%. They were maintained on a 12-h light/dark cycle, with lights on at 6:00 a.m. and off at 6:00 p.m. Before the experiments, the mice underwent a habituation period of at least 10 days in the animal facility while being fed a standard control diet.

Experiment 4 was performed using *Npy2r*-knockout (KO) mice. Germ line deletion of the *Npy2r* gene was achieved as previously described [24], [25]. The genotype of *Npy2r*-KO mice was confirmed with PCR. Mice were bred on a C57BL/6J background and housed in the same facility as the conventional C57BL/6J mice.

### Study design

Animals were fed either HFD (S5745-E712) containing 48% of energy from fat (20 wt% palm oil and 5 wt% soy oil) or a control diet (S5745-E702) consisting of 12% of energy from fat for eight weeks, a duration based on previous studies [5], [26]. Both diets contained 5% sucrose and were obtained from ssniff (Soest, Germany). Mice had ad libitum access to tap water and their respective diets, which were maintained throughout behavioral testing. Body weight was recorded weekly, and food intake per cage was assessed by measuring the change in food pellet weight. To facilitate comparison of food intake despite differences in diet composition, food intake was expressed in kilocalories (kcal) rather than grams. After the 8-week feeding period, the mice were subjected to the following experiments:

**Experiment 1** (*n* = 6 mice on control diet and 6 on HFD): Mice were subjected to the light/dark box test (LDT) on day 1 (D1) followed by the splash test on D2 (see Fig. 1a). Subsequently, locomotion, fluid intake, food intake, and sucrose preference (SP) were recorded over a 6-day period (D3–D8) in single-housed mice, starting with the beginning of the light cycle on D3, using the LabMaster system (TSE Systems, Bad Homburg, Germany; Fig. 1a) [27], [28].

**Fig. 1:**
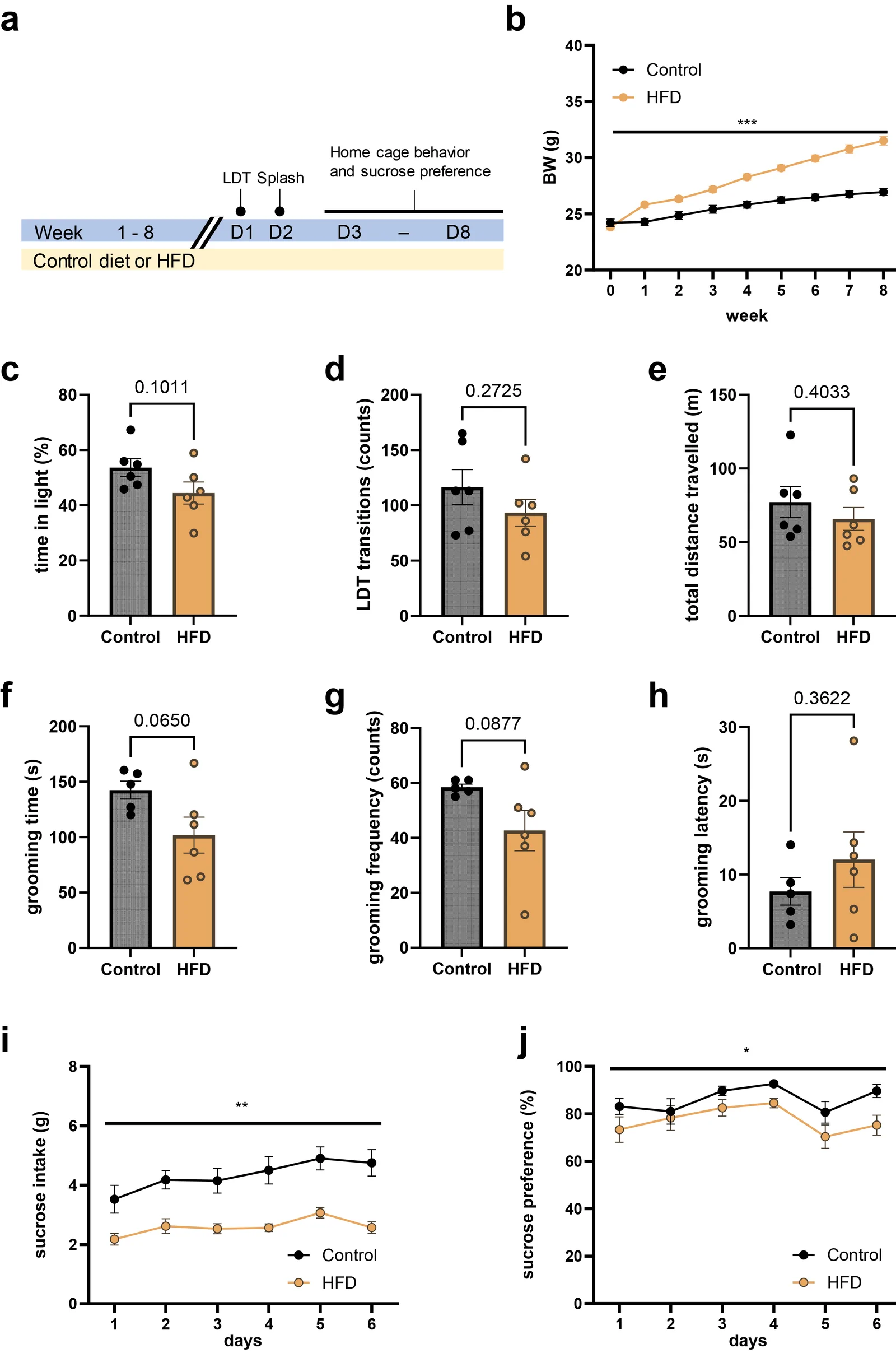
**Eight weeks of HFD-feeding leads to weight gain and decreased sucrose preference (SP).** Mice received a high-fat diet (HFD) or control diet for 8 weeks and were then subjected to the light/dark test (LDT), splash test and the SP test over 6 days (a). Body weight (BW) was assessed weekly (b). The LDT assessed the time spent in the light compartment (c), the number of transitions between light and dark compartment (d), as well as the distance traveled (e). The splash test assessed the time spent grooming (f), the number of grooming events (g), and the latency to the first groom (h). Sucrose intake (i) and SP (j) were assessed over 6 days within the automated home-cage monitoring platform (LabMaster). Values represent means ± SEM, *n* = 42 for BW of all mice from Experiments 1–3 and *n* = 5–6 for other panels; HFD main factor effect: ∗*p* ≤ 0.05, ∗∗*p* ≤ 0.01, ∗∗∗*p* ≤ 0.001.

**Experiment 2** (*n* = 12 mice on control diet and 12 on HFD): Mice were euthanized after the end of the 8-week feeding period. Heart blood was collected for the assessment of corticosterone, tryptophan, kynurenine, and cytokines. Colons were collected for the myeloperoxidase (MPO) assay.

**Experiment 3:** Mice on both control diet and HFD were subdivided to receive either intranasal (i.n.) distilled water as a vehicle or i.n. NPY resulting in the following four groups (*n* = 12 per group; Fig. 3a).

1. Control-H_2_O: Mice fed control diet and given i.n. distilled water as a vehicle
2. Control-NPY: Mice fed control diet and given i.n. NPY
3. HFD-H_2_O: Mice fed HFD and given i.n. distilled water as a vehicle
4. HFD-NPY: Mice fed HFD and given i.n. NPY

After HFD-feeding for 8 weeks, mice were singly housed for 5 days in standard cages in order to test the SP. Each cage contained two bottles: one with tap water and one with a 1% sucrose solution, allowing assessment of sucrose intake and SP. The position of the 2 bottles relative to each other was changed daily. I.n. NPY or vehicle was administered on days 2 and 4. Food, water, and sucrose intake were calculated based on weight changes according to the following schedule:

**Days 0**–**2:** Body weight, food intake, sucrose intake, and SP were measured once daily. Values collected on day 2 (prior to i.n. NPY/vehicle administration) served as baseline for assessing NPY effects at 24 h and 48 h post-administration.

**Day 3:** Measurements were taken once to assess the effect of i.n. NPY/vehicle 24 h post-administration.

**Day 4:** Body weight, food intake, sucrose intake, and SP were measured twice — once before i.n. NPY/vehicle administration [reflecting the long-term effect of i.n. NPY/vehicle after 48 hours (48 h)] and once 3 hours (3 h) after administration (reflecting short-term effects). These timepoints were selected based on previous studies which showed behavioral effects of intranasal NPY measured 3.5 h after administration of NPY [28] as well as after 24 h and 48 h [29], [30]. Mice that consumed less than 0.1 g total fluid in the 3 h SP test period were excluded from the analysis.

Following the second measurement on day 4, the mice were euthanized, blood plasma was collected for corticosterone measurement, and brains were collected to assess NPY expression at mRNA and peptide levels, as well as the mRNA expression of interleukin-1 beta (*Il1b*), glucocorticoid receptor (*Nr3c1)*, and corticotropin-releasing hormone (*Crh*) in the hypothalamus.

**Experiment 4:** To investigate the role of NPY2R in i.n. NPY-induced effects, *Npy2r*-KO mice were fed with HFD for 8 weeks before receiving either i.n. NPY or vehicle (Fig. 5a).

### Intranasal application of NPY

Mice were lightly sedated with isoflurane and a pipette was used to apply either 100 μg NPY (Phoenix Pharmaceuticals, Karlsruhe, Germany, catalog number 049-03) dissolved in 10 μL of sterile distilled water, or sterile distilled water only, equally divided between both nostrils of the sedated mice. After the application, the mice were held in a tilted backward position until they started to wake up to help NPY uptake from the nasal cavity to the brain. This dose of intranasal NPY was previously found to attenuate sickness behavior induced by lipopolysaccharide (LPS) [28].

### Behavioral tests

The LDT was utilized to evaluate anxiety-like behavior, while self-care behavior was measured through the splash test. The LabMaster system, designed for continuous monitoring in a specialized housing cage, tracked locomotion, exploration, drinking, feeding, and SP. Sucrose consumption and SP were used to evaluate sucrose-induced hedonic responses. The tests were performed as described previously [5], [6]. Due to the need for simultaneous i.n. administration and in order to minimize potential inter-batch variability, measurements of sucrose and water consumption, as well as food intake, in Experiments 3 and 4 were performed in standard cages by weighing sucrose and water bottles as well as food pellets before and after the specified measurement intervals according to published protocols [31].

### Collection of tissues and blood samples

Mice were deeply anesthetized by intraperitoneal pentobarbital injection (150 mg/kg). Blood was collected by cardiac puncture and centrifuged at 4100 x *g* for 15 min at 4 °C. After centrifugation, the blood plasma was immediately frozen on dry ice and stored at −70 °C until further use.

Collected brains were stored at −70 °C followed by microdissection of hypothalamus as described previously [28]. The microdissected hypothalamus was divided along the midline with one half being used for qPCR and the other for the NPY immunoassay.

To collect the colons for MPO assay, a 1 cm segment of the distal colon was cut open longitudinally, rinsed with saline, dried with tissue paper, and immediately frozen in liquid nitrogen.

### Ribonucleic acid (RNA) extraction and quantitative polymerase chain reaction (qPCR)

Hypothalamic tissue samples were homogenized in 1 mL QIAzol Lysis Reagent by using the Precellys Evolution Homogenizer from Bertin Technologies SAS (Montigny-le-Bretonneux, France). Then RNA was extracted with the RNeasy Lipid Tissue Mini Kit (QIAGEN) as described by the manufacturer.

Reverse transcription of extracted RNA to cDNA was performed using the High-Capacity cDNA Reverse Transcription Kit (Thermo Fisher Scientific). Gene expression was quantified using the TaqMan Gene Expression Assay (Thermo Fisher Scientific).

All samples were measured in triplicate and the relative quantification was conducted using the 2^−ΔΔCt^ method [32], with the mean expression of *Gapdh* (Mm99999915_g1) and *Ppil3* (Mm00510343_m1) serving as reference housekeeping genes. Group differences were expressed as fold changes relative to the control group. The analyzed targets included *Npy* (Mm03048253_m1), *Il1b* (Mm00434228_m1), *Nr3c1* (Mm00433832_m1), and *Crh* (Mm01293920_s1).

### NPY, MPO and corticosterone immunoassays

The concentration of NPY in the hypothalamus was measured using the NPY fluorescence immunoassay (Phoenix Pharmaceuticals, Burlingame, CA, USA) according to the manufacturer's instructions. NPY extraction was performed as previously described [5]. The peptide content was expressed relative to the protein content of the samples, which was measured using the BCA protein assay kit (Pierce Biotechnology, Rockford, IL, USA).

The concentration of MPO in the colon was analyzed using an enzyme immunoassay (EIA) kit specific for rat and mouse MPO proteins (Hycult Biotechnology, Uden, The Netherlands). Following homogenization of the colonic tissue, the assay was executed according to the manufacturer's instructions. The values were normalized to the tissue protein content, determined via the BCA protein assay kit (Pierce Biotechnology, Rockford, IL, USA).

Corticosterone plasma levels were determined with an EIA kit (Enzo Life Sciences, Farmingdale, NY, USA) according to the manufacturer's instructions.

### Plasma cytokines

Plasma levels of interleukin-6 (IL-6), interleukin-10 (IL-10), and tumor necrosis factor-alpha (TNF-α) were assessed using a ProcartaPlex Multiplex Immunoassay (Thermo Fisher Scientific, Waltham, MA, USA) according to the manufacturer's instructions. The assay was performed on the Bio-Plex 200 multiplex suspension array system and analyzed using Bio-Plex 5.0 software (Bio-Rad, Hercules, CA, USA).

### Kynurenine, kynurenic acid and tryptophan measurement

High-performance liquid chromatography (HPLC) with ultraviolet detection was used to quantify kynurenine, kynurenic acid and tryptophan in plasma samples, as previously described [27], [33].

### Statistical analysis

GraphPad Prism 10.5.0 (GraphPad Software Inc., La Jolla, CA, USA) was used for statistical analysis and creation of the graphs. Independent *t*-tests, two-way ANOVA, and mixed-effects models were performed as appropriate. For the two-way ANOVA, the factors included diet (HFD vs. control diet) and intranasal treatment (NPY vs. vehicle). Data distribution was assessed using the Shapiro-Wilk test in combination with visual inspection of Q-Q plots. Homoscedasticity was assessed by F-test of equality of variances as well as visual inspection of residual plots. For comparisons between two independent groups, Welch's correction was used when variances were unequal. Where the Shapiro–Wilk test and inspection of the Q-Q plots indicated a clear departure from normality, the two groups were compared with the Mann–Whitney *U* test instead of a *t*-test. Data were transformed where necessary to satisfy the assumptions of the statistical model; with the specific transformations being indicated in the corresponding figure legends. For repeated-measures analyses, the Greenhouse-Geisser correction was applied to correct for violations of sphericity. For post hoc multiple comparisons, Šídák's correction was used for pairwise comparisons within the two-group repeated-measures factors, whereas Tukey's correction was used for pairwise comparisons following analyses involving 4 groups. A *p*-value ≤0.05 was considered statistically significant. Group sizes were based on our previous studies employing comparable experimental designs and behavioral endpoints [5], [28]. Data are presented as mean ± SEM.

## Results

### HFD leads to weight gain and modest behavioral changes

Mice on HFD gained more weight than those on the control diet (F(1, 82) = 38.56, *p* < 0.001) (Fig. 1b). No significant effects of HFD on anxiety-like behavior were observed in the LDT, while the splash test showed a non-significant trend toward reduced grooming behavior (Fig. 1c–h, Table S1). Mice on HFD showed a clear, statistically significant reduction in sucrose intake (F(1,8) = 18.6, *p* = 0.003) (Fig. 1i) and a modest but statistically significant reduction in SP (F(1,8) = 5.9, *p* = 0.040) (Fig. 1j) when tested over 6 days in the automated home-cage monitoring platform (LabMaster).

Daily activity assessed across the light and dark phases or solely the dark phases did not reveal a significant effect of HFD during the 6-day observation period (Fig. 2a–d, Table S1). However, both horizontal (F(1,8) = 6.1, *p* = 0.038) and vertical activity (F(1,8) = 7.9, *p* = 0.023) during the light cycle showed a significant decrease by HFD (Fig. 2 e,f). Again, no significant diet × time interaction was detected (Table S1). In summary, automated home-cage monitoring revealed a reduction in sucrose-directed ingestive behavior and a reduction in locomotor activity that specifically reached significance during the light cycle in mice on HFD.

**Fig. 2:**
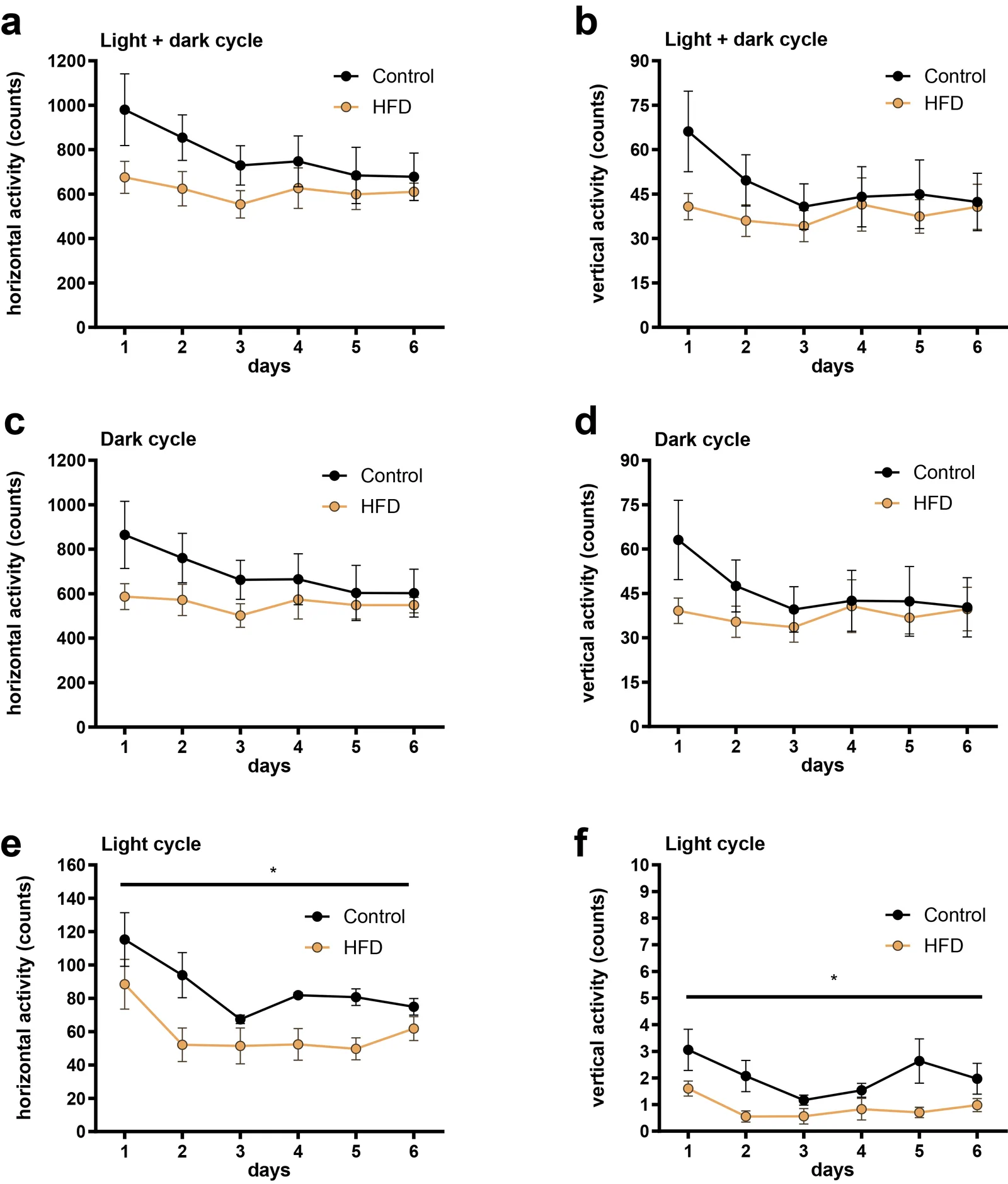
**Eight weeks of HFD-feeding decreases home cage activity.** Mice were fed a high-fat diet (HFD) or control diet for 8 weeks. Locomotor activity was then assessed using an automated home-cage monitoring system (LabMaster) over 6 days. Horizontal (a, c, e) and vertical activity (b, d, f) is shown over 24 h (a, b), as well as during the dark cycle (c, d), and during the light cycle (e, f). Values represent means ± SEM, *n* = 5; HFD main factor effect: ∗*p* ≤ 0.05.

**Fig. 3:**
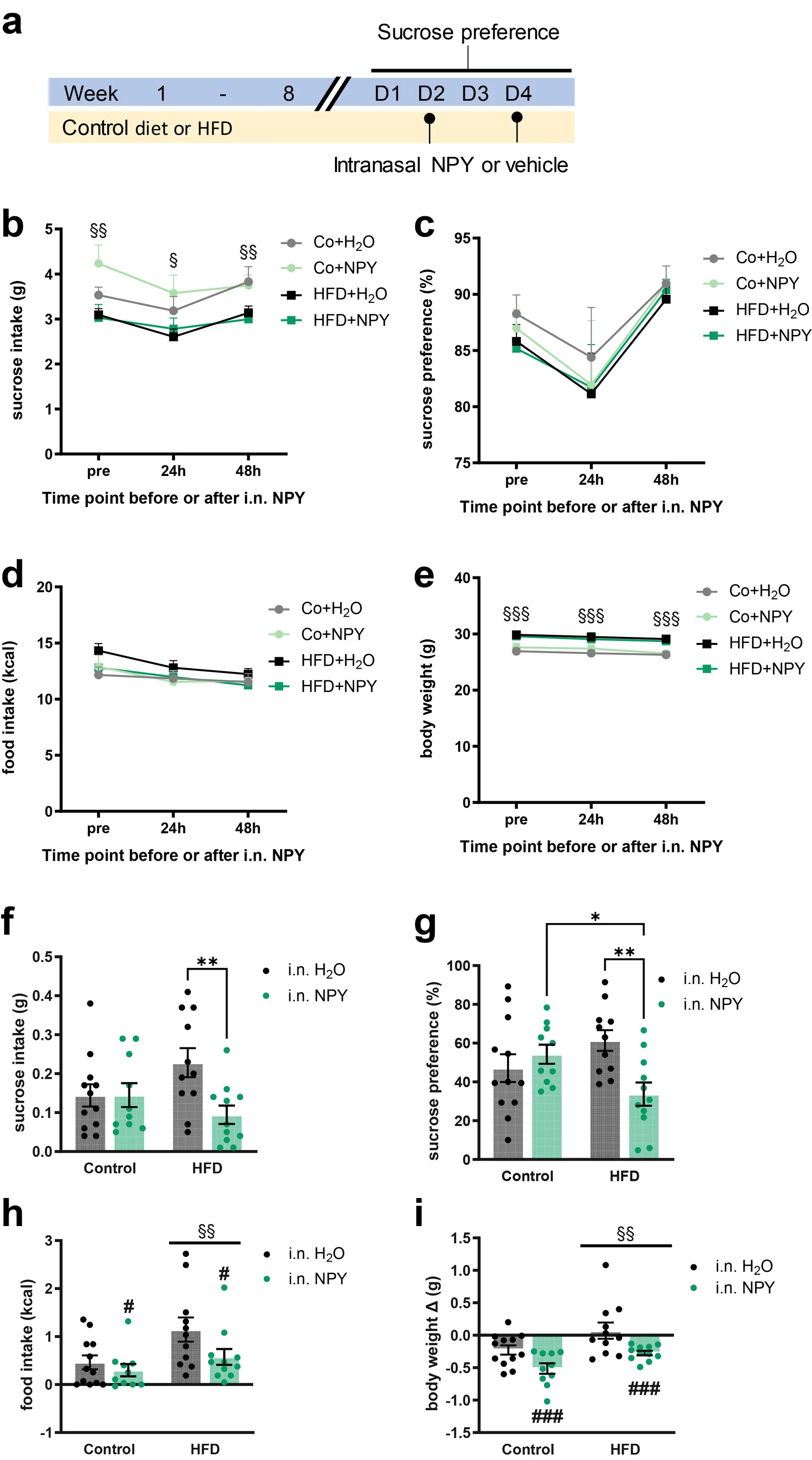
**Intranasal NPY leads to diet-dependent decreases in ingestive behaviors.** Mice were fed a high-fat diet (HFD) or control (Co) diet for 8 weeks followed by intranasal (i.n.) administration of NPY or vehicle (H_2_O) on days 2 (D2) and 4 (D4) (a). Sucrose intake (b, f), sucrose preference (SP) (c, g), food intake (d, h), and body weight (e, i) were assessed 3 h (f–i), as well as 24 h and 48 h (b–e) after NPY administration. Analysis was performed on log transformed data for 3 h sucrose intake using log_10_(Y) and 3 h food intake using log_10_(Y+1) to meet assumptions. Values represent means ± SEM, *n* = 9–12; multiple comparisons in case of a significant interaction: ∗*p* ≤ 0.05, ∗∗*p* ≤ 0.01; HFD main factor effect: ^§^*p* ≤ 0.05; ^§§^*p* ≤ 0.01; ^§§§^*p* ≤ 0.001; NPY main factor effect: ^#^*p* ≤ 0.05; ^###^*p* ≤ 0.001.

### HFD reduces basal plasma corticosterone levels

Mice on HFD showed significantly lower basal plasma corticosterone levels (t _(13.8)_ = 3.54, *p* = 0.003, 95% CI: -19.45 to −4.763) than mice on control diet. No significant differences could be identified in response to diet in plasma levels of TNF-α, IL-6, IL-10, tryptophan, kynurenine, kynurenine-to-tryptophan ratio, or kynurenic acid. Similarly, colonic MPO did not significantly differ in response to diet (Table 1).

**Table 1:** Effects of HFD on circulating corticosterone, cytokines, and tryptophan–kynurenine pathway metabolites, and on colonic myeloperoxidase (MPO).

	Control	HFD	*p-*value Effect size	95% CI/Hodges–Lehmann interval
Corticosterone (ng/mL) *n* = 12	16.11 ± 3.22	**4.01 ± 1.15**	**0.0033** η^2^ = **0.477**	**−19.45 – −4.76**
TNF-α (pg/mL) *n* = 8–9	3.28 ± 0.70	2.62 ± 0.78	0.5382 η^2^ = 0.026	−2.88 – 1.57
IL-6 (pg/mL) *n* = 8–9	5.14 ± 0.87	3.24 ± 0.73	0.1188 η^2^ = 0.154	−4.35 – 0.55
IL-10 (pg/mL) *n* = 8–9	4.33 ± 0.54	3.31 ± 0.66	0.2469 η^2^ = 0.088	−2.83 – 0.79
Tryptophan (μM) *n* = 11–12	43.78 ± 2.60	39.92 ± 2.64	0.3101 η^2^ = 0.049	−11.59 – 3.86
Kynurenine^†^ (μM) *n* = 11–12	0.52 (0.43–0.63)	0.37 (0.35–0.59)	0.0997 *r* = 0.409	−0.18 – 0.04
Kynurenine/tryptophan ratio^†^ (× 10^−3^) *n* = 11–12	10.66 (9.18–15.68)	10.20 (8.97–14.31)	0.6075 *r* = 0.136	−3.62 – 2.13
Kynurenic acid (nM) *n* = 11–12	42.27 ± 4.98	42.13 ± 4.59	0.9845 η^2^ = 0.000	−14.19 – 13.92
MPO colon^†^ (ng/mg) *n* = 12	0.07 (0.02–0.16)	0.09 (0.04–0.11)	0.*9661* *r* = *0*.*01*4	−0.06 – 0.06

Mice were fed a control diet or HFD for 8 weeks, and parameters were measured at the end of the experiment. Statistically significant differences are shown in bold. Parameters marked † are presented as median (25th–75th percentile) and were analyzed with the exact Mann–Whitney *U* test; for these, effect sizes are given as rank-biserial correlation (r) and the intervals are Hodges–Lehmann intervals for the median of pairwise differences. All other parameters are presented as mean ± SEM and were analyzed with an independent-samples *t*-test; for these, effect sizes are given as η^2^ and the intervals are 95% CIs for the difference between means.

### Intranasal NPY does not affect ingestive behavior or body weight at 24 h or 48 h post-administration

Given that i.n. NPY has been demonstrated to elicit long-term behavioral effects [29], [30], potential effects of i.n. NPY on ingestive behavior were investigated 24 h and 48 h after i.n. NPY administration. In Experiment 3, manual assessment of sucrose intake at baseline, 24 and 48 h after NPY or vehicle administration confirmed the pattern observed in the automated home-cage monitoring results. HFD-fed mice showed significantly lower absolute sucrose intake than control-fed mice at all time points [baseline F(1,38) = 8.8, *p* = 0.005; 24 h F(1,38) = 5.4, *p* = 0.026; 48 h F(1,37) = 9.1, *p* = 0.005] (Fig. 3b, Table S1). HFD was also associated with a modest reduction in SP, resembling the pattern observed in Experiment 1, although this effect did not reach statistical significance (Fig. 3c, Table S1). Compared with i.n. vehicle, i.n. NPY did not affect sucrose intake or SP at these time points, and no diet × treatment interaction was observed (Table S1). Total food intake did not differ between diet groups at baseline, and neither diet nor treatment affected intake 24 or 48 h after i.n. NPY/vehicle administration. No diet × treatment interaction was observed (Fig. 3d, Table S1). Body weight was significantly increased by HFD, with no effect of i.n. treatment, and no diet × treatment interaction (Fig. 3e, Table S1).

### Intranasal NPY causes short-term reductions in sucrose consumption, food intake, and body weight 3 h post-administration

In contrast to the absence of significant i.n. treatment effects at 24 h and 48 h, i.n. NPY produced clear short-term effects 3 h after administration, affecting sucrose intake and SP, as well as caloric food intake and body weight (Fig. 3f–i). For sucrose intake, two-way ANOVA revealed a significant diet × treatment interaction (F(1,40) = 5.5, *p* = 0.024), and post hoc analysis showed that i.n. NPY significantly reduced sucrose intake only in the HFD group (*p* = 0.002, 95% CI: 0.1893–0.8004) (Fig. 3f, Table S1). This effect was mirrored by a marked reduction in SP: at the 3 h time point, SP remained at 45–60% in the Control-H_2_O, Control-NPY, and HFD-H_2_O groups, whereas it dropped to ∼30% in the HFD-NPY group (Fig. 3g). Consistently, two-way ANOVA revealed a significant diet × treatment interaction for SP (F(1,40) = 8.3, *p* = 0.006), and post hoc analysis confirmed that i.n. NPY significantly reduced SP in HFD mice (*p* = 0.002, 95% CI: 10.48–44.91), but not in control-diet-fed mice (*p* = 0.408, 95% CI: -24.45 - 10.13) (Fig. 3g).

Beyond these effects on sucrose-related measures, diet and i.n. treatment also influenced caloric food intake and body weight (Table S1). HFD-fed mice consumed more calories (F(1,40) = 8.3, *p* = 0.006, Table S1) and lost less weight (F(1,40) = 10.09, *p* = 0.003) 3 h after i.n. treatment than control-diet-fed mice. Across diet groups, 3 h after the application, i.n. NPY reduced food intake (F(1,40) = 4.1, *p* = 0.049) and enhanced weight loss (F(1,40) = 13.9, *p* < 0.001) compared with i.n. H_2_O, with no significant diet × treatment interaction for either measure (Fig. 3h and i, Table S1).

### HFD reduces the expression of NPY in the hypothalamus

Both NPY peptide concentrations (F(1,36) = 9.7, *p* = 0.004) and mRNA expression (F(1,36) = 29.3, *p* < 0.001) were reduced in the hypothalamus of HFD-fed mice (Fig. 4a and b, Table S1). *Il1b* mRNA showed a trend to increase in response to HFD (F(1,37) = 3.7, *p* = 0.062) (Fig. 4c), but was not affected by i.n. NPY treatment, and no significant diet × treatment interaction was detected (Fig. 4c,Table S1).

**Fig. 4:**
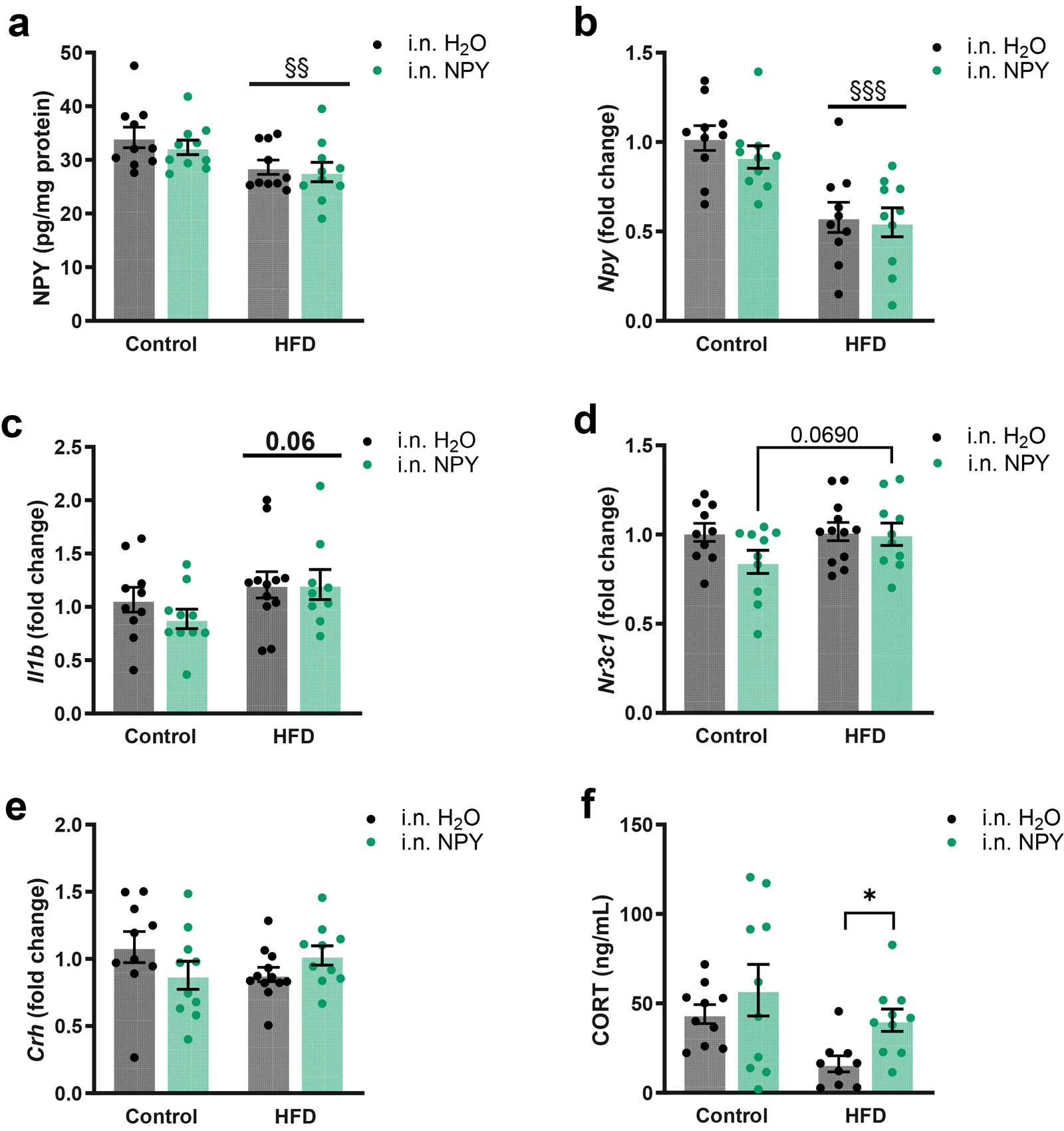
**Effects of HFD and intranasal NPY on hypothalamic neuroendocrine and immune markers as well as circulating corticosterone.** Mice were fed a high-fat diet (HFD) or control diet (Co) for 8 weeks and received intranasal (i.n.) administration of NPY or vehicle (H_2_O) followed by plasma and brain collection 3 h after the second administration of NPY. Hypothalamic concentration of NPY (a). Relative expression of *Npy* (b), *Il1b* (c), the glucocorticoid receptor *Nr3c1* (d), and corticotropin-releasing hormone (*Crh*) (e) mRNA in hypothalamus. Plasma concentration of corticosterone (f). Analysis was performed on log transformed data for circulating corticosterone using the function log_10_(Y) to meet assumptions. Values represent means ± SEM, *n* = 9–12; multiple comparisons in case of a significant interaction: ∗*p* ≤ 0.05, ∗∗*p* ≤ 0.01; HFD main factor effect: ^§^*p* ≤ 0.05; ^§§^*p* ≤ 0.01, ^§§§^*p* ≤ 0.001.

**Fig. 5:**
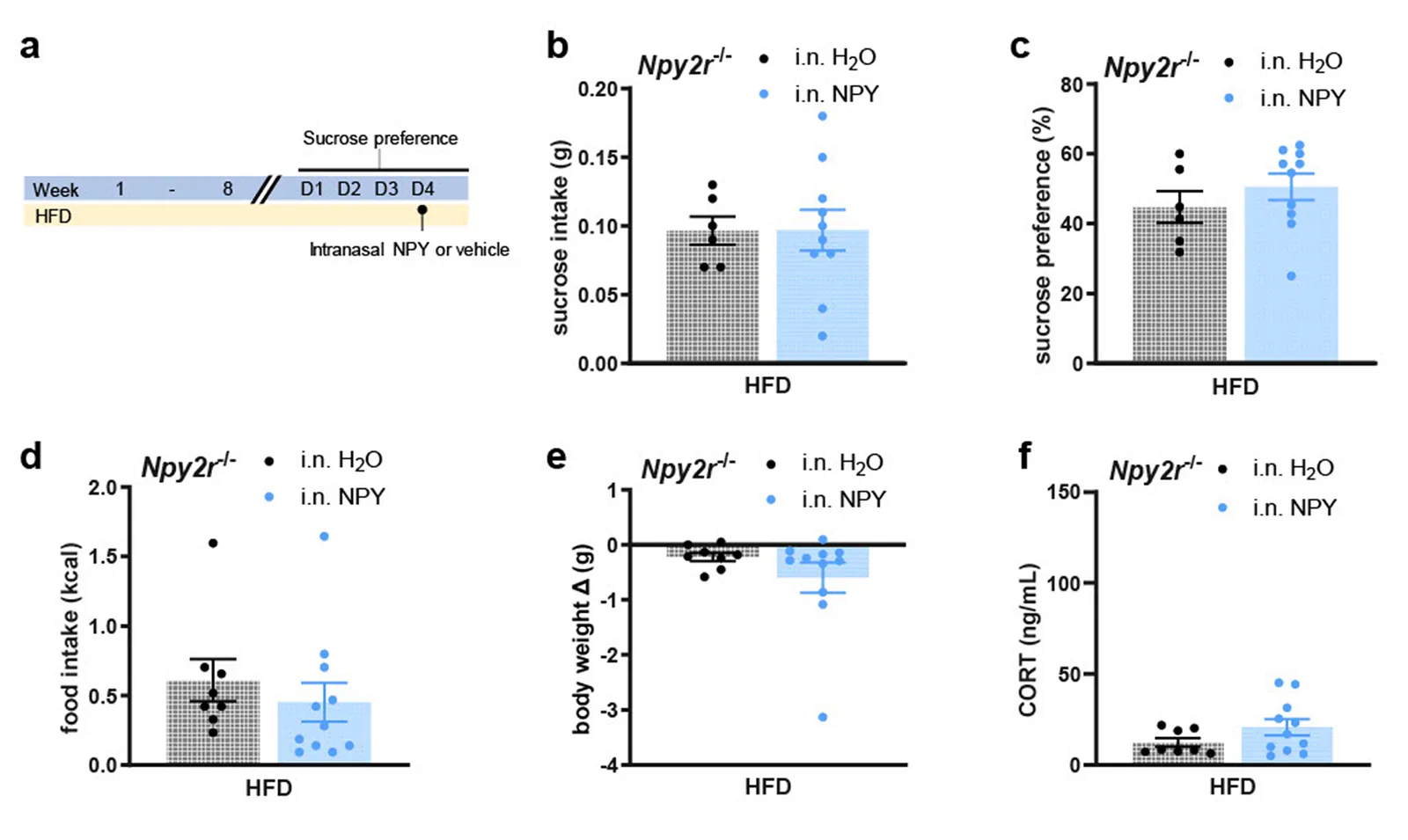
**Effects of intranasal NPY on ingestive behavior and circulating corticosterone are attenuated in *Npy2r*-deficient mice on HFD.***Npy2r*-deficient mice were fed a high-fat diet (HFD) for 8 weeks and the sucrose preference (SP) test was performed subsequently between day 1 (D1) and day 4 (D4) (a). Intranasal (i.n.) NPY or vehicle (H_2_O) was administered on D4 and sucrose intake (b), sucrose preference (SP) (c), food intake (d), body weight change (e), and circulating corticosterone (CORT) levels (f) were assessed 3 h after NPY administration. Analysis was performed on log transformed data using the function log_10_(Y) for food intake and log_10_(0.2 - Y) for body weight to meet assumptions. Values represent means ± SEM, *n* = 6–11.

### HFD and NPY influence circulating corticosterone

While the mRNA expression of hypothalamic *Crh* and *Nr3c1* showed no statistically significant differences across the groups (Fig. 4d and e, Table S1), HFD reduced circulating corticosterone (F(1,35) = 4.7, *p* = 0.038), which is consistent with the finding of Experiment 2 (Table 1). In addition, there was a significant diet × treatment interaction for corticosterone (F(1,35) = 5.6, *p* = 0.024), and post hoc analysis confirmed that i.n. NPY increased corticosterone levels in HFD-fed mice (*p* = 0.02), but not in control diet-fed mice (*p* = 0.67) (Fig. 4f).

### *Npy2r*-KO mice do not exhibit changes in SP, food consumption, body weight, or circulating corticosterone following i.n. NPY administration

Since the observed effects of i.n. NPY on food intake and SP are consistent with NPY2R-mediated effects, we examined the effect of i.n. NPY administration in *Npy2r*-KO mice in Experiment 4 (Fig. 5a). Unlike wild type (WT) C57BL/6J mice in Experiment 3, in Experiment 4 *Npy2r*-KO mice on HFD showed no changes in sucrose intake, SP, food intake, body weight, or circulating corticosterone levels 3 h after i.n. NPY administration (Fig. 5b–f, Table S1). These findings are consistent with a potential involvement of NPY2R in the anorexigenic and reward-modulating effects of i.n. NPY.

## Discussion

This study investigated the effects of HFD containing fat levels representative of Western dietary patterns [23] on behavior and selected stress-related signaling pathways in mice. We further aimed to elucidate whether i.n. NPY administration modulates the observed outcomes. Our findings show that a Western-like HFD produced a robust reduction in sucrose intake and a modest decrease in SP. These behavioral changes were accompanied by reduced circulating corticosterone levels and decreased hypothalamic expression of NPY. Unexpectedly, in HFD-fed mice, i.n. NPY reduced short-term sucrose intake and SP, and increased circulating corticosterone. Independently of diet, i.n. NPY also decreased food intake and body weight. In contrast, i.n. NPY failed to alter sucrose intake, SP, food intake, body weight, or circulating corticosterone in HFD-fed *Npy2r*-KO mice, consistent with a potential involvement of NPY2R in the observed effects of NPY.

In the present study, HFD did not affect anxiety-like behavior, as assessed by the LDT. These findings are consistent with our previous work using a higher concentration of HFD [5]. Behavioral changes induced by HFD in rodents have been widely reported; however, the effect of HFD on anxiety-like behavior remains inconsistent across studies, with anxiogenic effects, anxiolytic effects, and no effects of HFD on anxiety-like behaviors being reported [34], [35], [36]. The variability in behavioral alterations induced by HFD likely reflects differences in HFD composition and duration, as well as age at exposure [37], [38], [39]. Notably, a reduction in the hedonic response to sucrose reflected by decreased sucrose intake emerged as a reproducible finding in our laboratory across two HFD concentrations (48% and 60% of calories from fat, present study and [5]), and has also been consistently reported by independent laboratories using diverse experimental paradigms [40], [41]. For i.n. NPY administration, we used a well-established protocol previously applied in our laboratory and by other groups [28], [29], [42]. The use of distilled water as a vehicle is based on the majority of previous preclinical studies and recommendations, as NPY has greater solubility in water [43]. On the other hand, isotonic solutions may be more physiologically suitable for the respiratory epithelium, particularly with repeated administration and at higher administration volumes. The fixed absolute dose and volume used in this study, without normalization to individual mouse body weight, were chosen because our aim was to achieve nose-to-brain delivery rather than systemic exposure. This approach is also consistent with previous preclinical and clinical studies of intranasal NPY using fixed doses [22], [29], [44]. An approach that may enhance the efficacy of i.n. NPY, but was not incorporated into our study, is coadministration with a dipeptidyl peptidase IV (DPP-IV) inhibitor to reduce the enzymatic degradation of NPY. Nahvi et al. (2021) demonstrated that this approach can enhance the efficacy of otherwise subtherapeutic doses of i.n. NPY, rendering them effective [22]. Despite these promising findings, DPP-IV inhibition has not yet been widely adopted in i.n. NPY research.

As in other studies, the procedure itself involved several potential stress-related confounders, including anesthesia, single housing, and the intranasal administration procedure. While a possible interaction between NPY and procedure-related stress cannot be ruled out completely, NPY has been demonstrated to ameliorate stress-induced as well as inflammation-induced behavioral disturbances already 2–3.5 h after i.n. administration [28], [29]. It is noteworthy that SP in our study was low when assessed 3 h after i.n. treatment (≈30% in the HFD-NPY group and ≈50% in the other groups) but markedly higher (>80%) when measured after 24 h. This pattern is consistent with the computational analyses of SP behavior in mice, which demonstrate that SP stabilizes progressively over the course of testing [45]. Importantly, despite the relatively low SP 3 h after i.n. treatment, NPY exerted a clear and statistically significant effect on SP in HFD-fed mice (Fig. 3g). The reduction in SP following i.n. NPY has been reported previously in rats receiving a smaller dose of NPY (100 μg/kg), supporting the consistency of this effect across the two species [30]. Interestingly, in the same study, i.n. NPY reduced the immobility time in the forced swim test [30]. The SP test is a well-established paradigm for assessing sucrose-related hedonic behavior; however, anhedonia is a multidimensional construct, and the SP test alone is insufficient to reflect generalized anhedonia especially in the presence of nutritional confounders [46]. Alterations in other aspects of reward-related behavior have also been reported in response to high-calorie diets. For example, in obese rats reduced responsiveness to sexual cues in the female urine sniffing test and higher intracranial self-stimulation thresholds have been reported, indicating impaired brain reward function [47], [48]. Thus, high-calorie diets may affect multiple dimensions of reward-related behavior, whereas the present study specifically focused on sucrose-related hedonic behavior in relation to NPY. Whether NPY also influences other dimensions of reward dysfunction remains to be investigated.

Behavioral alterations in DIO are associated with dysregulated signaling pathways that intersect with neurobiological mechanisms implicated in reward dysfunction and depression [8]. In the present study, we focused on systemic and central inflammatory signaling, IDO pathway activation, HPA axis regulation, and NPY signaling. Whereas circulating cytokines, colonic MPO levels, hypothalamic *Il1b* mRNA expression, and the kynurenine-to-tryptophan ratio were not significantly affected by HFD, circulating corticosterone levels and hypothalamic NPY signaling were significantly reduced in response to HFD feeding. Notably, the effects of HFD on circulating corticosterone, circulating cytokines, and IDO activity are inconsistent across studies, with reductions, no change, and increases all reported [5], [49], [50], [51], [52], [53], [54], [55]. However, we did not evaluate all aspects of inflammation and did not specifically assess IDO activity in the brain or peripheral tissues. Therefore, a subtle inflammatory response or tissue-specific activation of the IDO pathway cannot be completely excluded. In contrast, DIO in our study was associated with a suppression of hypothalamic NPY signaling, consistent with multiple independent reports, including our prior work using a higher concentration of HFD and earlier studies [5], [56], [57], supporting the reproducibility of this finding.

NPY can modify mood and behavior because of its anxiolytic, orexigenic, and antidepressant effects, and its interaction with the HPA axis [58]. Therefore, we hypothesized that i.n. NPY may improve HFD-induced behavioral impairments. Contrary to expectations, i.n. NPY lowered sucrose intake and SP in the HFD group 3 h after application. These findings were accompanied by lower food intake and weight loss after i.n. NPY application, a pattern consistent with NPY2R-mediated effects rather than NPY1R. While NPY1R activation promotes carbohydrate intake and increases meal size, NPY2R activation exerts an opposing, inhibitory influence on feeding behavior [10], [59].

The NPY2R is the most abundant subtype of NPY receptors in the CNS [60]. Moreover, NPY_1-36_, which can operate via NPY1R, NPY2R, and NPY5R, exhibits a short plasma half-life largely due to rapid cleavage by DPP-IV [61]. In one human study, the plasma half-life of NPY_1-36_ was 10.1 min, whereas its major degradation product, NPY_3-36_, is considerably more stable, with a half-life of approximately 27.3 min [61]. NPY_3-36_ has a 1000-fold lower affinity for NPY1R but retains the same affinity for NPY2R as compared to NPY_1-36_ and therefore selectively activates NPY2R and NPY5R [62]. The degradation of NPY_1-36_ to NPY_3-36_ does not occur exclusively in plasma but can also occur at the nasal mucosa and within brain tissue, as both tissues exhibit DPP-IV-like activity [63], [64]. This is particularly relevant for intranasal NPY studies: early experiments using immunofluorescently labeled intranasal NPY confirmed peptide-associated signals in brain regions including the hypothalamus and hippocampus [65]; however, this approach did not distinguish whether the detected signal represented intact parent NPY_1-36_ or proteolytic metabolites. Therefore, proteolytic conversion of NPY before receptor engagement within the brain cannot be excluded. Future studies are warranted to explore the proposed mechanism in more detail, analyze peptide processing and NPY metabolites *in vivo*, investigate receptor activation by NPY metabolites, track brain delivery and peptide distribution within the brain, as well as assess central bioavailability and the anatomical site of action after i.n. administration of NPY.

Given the anorexigenic effect of i.n. NPY 3 h post-administration, we hypothesized that NPY2R might be potentially involved. To test this hypothesis, we examined the effect of i.n. NPY in *Npy2r*-KO mice. The finding that i.n. NPY has no effect on sucrose intake, SP, food intake, body weight, and circulating corticosterone in *Npy2r*-KO mice is consistent with a potential involvement of NPY2R. Importantly, however, baseline food intake, sucrose intake, and SP in *Npy2r*-KO mice on HFD were lower than in WT mice on HFD. While this phenotype might appear counterintuitive, it aligns with previous reports of an altered circadian control of locomotion and ingestive behaviors in these mice [66]. Therefore, it cannot be ruled out that the failure of i.n. NPY to further reduce food intake in *Npy2r*-KO mice on HFD is due to a floor effect.

While the i.n. route of NPY administration has been proposed as a feasible approach to treat brain disorders in both rodents and humans [44], [67], the effect of this approach on food intake and differential food preference remains less investigated. The results of our study address the necessity of thoroughly investigating a wide range of physiological actions of i.n. administered NPY, as the overall effect of i.n. NPY may differ from that of local injection of the peptide in specific brain regions.

In our study, no significant effects of i.n. NPY were detected 24 or 48 h post-administration. This absence of sustained effects following i.n. NPY is likely to reflect the short biological half-life of the peptide and is consistent with a report describing a lack of long-term effects of i.n. NPY on food intake and body weight [68]. Although behavioral effects of NPY have been reported at 24 and 48 h after administration [29], [30], these effects may reflect a protective effect against the subsequent consequences of acute stress paradigms applied shortly after NPY administration and do not necessarily indicate a persistent direct effect of NPY at these later time points.

In the present study, DIO was associated with reduced circulating corticosterone. Several mechanisms may explain this finding, including attenuation or diurnal reorganization of central HPA-axis activity, reduced adrenal responsiveness to ACTH due to a mechanism potentially involving hyperleptinemia, and altered peripheral glucocorticoid metabolic clearance [49], [69], [70]. Moreover, our results suggest a possible role for NPY, since reduced hypothalamic NPY was accompanied by lower circulating corticosterone, whereas i.n. NPY increased circulating corticosterone in WT obese mice but not in *Npy2r*-KO obese mice. This pattern is consistent with the positive associations between hypothalamic NPY expression or peptide content and circulating corticosterone reported in rats under specific circadian and dietary conditions [71], [72].

The impact of exogenous NPY on the HPA axis appears to depend on both the route of administration and preexisting physiological conditions. While i.n. NPY counteracts the reactivity of the HPA axis to psychological stress as well as LPS injection [28], [58], central administration of NPY in the absence of preexisting stress is often associated with increased HPA-axis activity. For example, NPY administration into the paraventricular nucleus (PVN) increases circulating adrenocorticotropic hormone (ACTH) and corticosterone levels in rats [73]. Douglass et al. (2023) showed that fasting activates Agouti-related peptide (AgRP) neurons in the hypothalamus of mice, which activate the HPA axis and lead to higher corticosterone levels in the blood plasma through a mechanism that involves both NPY2R activation and GABA [74]. In the current study, i.n. NPY increased circulating corticosterone in WT but not in *Npy2r*-KO mice on HFD, which is consistent with the aforementioned studies conducted in the absence of preexisting stress.

Taken together, a Western-type HFD reduces sucrose intake, SP, hypothalamic NPY, and circulating corticosterone levels. I.n. NPY reduces short-term sucrose intake and SP but increases circulating corticosterone levels specifically in HFD-fed mice and reduces short-term food intake and body weight independently of diet. The effects of i.n. NPY on sucrose and food intake as well as on circulating corticosterone are absent in *Npy2r*-KO mice, consistent with a potential involvement of NPY2R. The acute nature of the study, the exclusive use of male mice, and the absence of a chronic treatment paradigm limit the translational interpretation of the current findings. Our data emphasize that the expanding use of i.n. NPY in experimental and clinical studies warrants careful analysis of the metabolism, pharmacokinetics, and receptor mechanisms of intranasally administered peptides.

## Author contributions

M.H., A.M.H. and A.F. performed the experiments and drafted the manuscript. M.H., K.H., and A.F. prepared and revised figures. K.H. and C.K.I. assisted with data analysis and interpretation. F.R. and A.J. assisted with the experiments. H.H. provided the *Npy2r*-KO mouse model. A.M. was responsible for the laboratory measurements of kynurenine, tryptophan, and kynurenic acid. P.H. contributed to the study design and acquired funding. A.M.H. and A.F. designed and supervised the study. All authors reviewed and approved the final manuscript.

## Declaration of generative AI and AI-assisted technologies in the writing process

Artificial intelligence (ChatGPT, OpenAI; Claude, Anthropic; and DeepL, DeepL SE) was used solely to enhance the language and readability of the manuscript; no graphs or results were generated by these tools. After using these tools, the authors reviewed and edited the content as needed and take full responsibility for the content of the published article.

## Declaration of competing interest

The authors declare that they have no known competing financial interests or personal relationships that could have appeared to influence the work reported in this paper.
