# Supplementary material for: Intranasal neuropeptide Y suppresses food intake, reduces sucrose preference and increases circulating corticosterone in obese mice
Source: Neurotherapeutics. 2026 Oct 22;24(1):e01100. doi: 10.1016/j.neurot.2026.e01100 (PMC13718799; doi:10.1016/j.neurot.2026.e01100)
Supplement: Supplementary file 1 [file mmc1.docx]

**Supplementary Table S1.** Statistical details of the effects of high-fat diet (HFD) and intranasal neuropeptide Y (NPY) on behavioural, metabolic and molecular outcomes.

| **Outcome** | **Effect** | ***t*(df) / *F* (df)** | ***p*** | **95% CI** | **effect size** |
| --- | --- | --- | --- | --- | --- |
|  |  |  |  |  | **η²_p_** |
| Body weight (Fig 1) | HFD | 38.56 (1, 82) | <0.001 | -3.22, -1.66 | 0.3198 |
| Sucrose intake (Fig 1) | HFD | 18.62 (1, 8) | 0.003 | 0.81, 2.68 | 0.6995 |
| Sucrose preference (Fig 1) | HFD | 5.977 (1, 8) | 0.040 | 0.49, 16.94 | 0.4276 |
| **Light–dark test (Fig 1) η²** | | | | | |
| Time in light | HFD | 1.81 (10) | 0.101 | −2.15, 20.54 | 0.246 |
| Transitions | HFD | 1.16 (10) | 0.273 | −21.29, 67.62 | 0.119 |
| Distance travelled | HFD | 0.87 (10) | 0.403 | −17.66, 40.40 | 0.071 |
| **Splash test (Fig 1)** | | | | | |
| Grooming time | HFD | 2.10 (9) | 0.065 | −3.13, 84.57 | 0.329 |
| Grooming frequency | HFD | 2.11 (5.25) Welch-corrected | 0.086 | −3.18, 34.64 | 0.459 |
| Grooming latency | HFD | 0.96 (9) | 0.362 | −14.46, 5.84 | 0.093 |
| **Home-cage activity (Fig 2)** | | | | | |
| Horizontal activity, light + dark cycle | HFD | 1.65 (1, 8) | 0.235 | −130.6, 458.5 | 0.131 |
|  | HFD × time | 2.42 (2.7, 21.8) | 0.099 | – | 0.034 |
| Vertical activity, light + dark cycle | HFD | 0.71 (1, 8) | 0.423 | −16.50, 35.58 | 0.063 |
|  | HFD × time | 2.95 (3, 23.9) | 0.053 | – | 0.045 |
| Horizontal activity, dark cycle | HFD | 1.23 (1, 8) | 0.299 | −148.4, 423.6 | 0.105 |
|  | HFD × time | 2.58 (3, 24.4) | 0.076 | – | 0.036 |
| Vertical activity, dark cycle | HFD | 0.54 (1, 8) | 0.485 | −17.92, 34.60 | 0.049 |
|  | HFD × time | 3.05 (3, 23.8) | 0.048 | – | 0.044 |
| Horizontal activity, light cycle | HFD | 6.14 (1, 8) | 0.038 | 1.82, 50.93 | 0.228 |
|  | HFD × time | 1.21 (2.1, 16.7) | 0.324 | – | 0.030 |
| Vertical activity, light cycle | HFD | 7.87 (1, 8) | 0.023 | 0.21, 2.19 | 0.244 |
|  | HFD × time | 0.91 (1.6, 12.8) | 0.406 | – | 0.038 |
| **Long-term (24 and 48 h) ingestive behaviour and body weight (Fig 3)** | | | | | |
| Sucrose intake 24 h | HFD | 5.39 (1, 38) | 0.026 | 0.09, 1.28 | 0.120 |
|  | NPY | 0.92 (1, 38) | 0.344 | -0.88, 0.31 | 0.020 |
|  | HFD × NPY | 0.13 (1, 38) | 0.719 | -1.41, 0.98 | 0.003 |
| Sucrose intake 48 h | HFD | 9.05 (1, 37) | 0.005 | 0.24, 1.21 | 0.196 |
|  | NPY | 0.22 (1, 37) | 0.644 | -0.37, 0.60 | 0.005 |
|  | HFD × NPY | 0.02 (1, 37) | 0.896 | -1.03, 0.91 | 0.000 |
| Sucrose preference 24 h | HFD | 0.15 (1, 38) | 0.704 | -7.46, 10.93 | 0.004 |
|  | NPY | 0.04 (1, 38) | 0.841 | -8.28, 10.11 | 0.001 |
|  | HFD × NPY | 0.11 (1, 38) | 0.740 | -15.35, 21.43 | 0.003 |
| Sucrose preference 48 h | HFD | 0.75 (1, 37) | 0.391 | -1.28, 3.21 | 0.020 |
|  | NPY | 0.14 (1, 37) | 0.715 | -2.66, 1.84 | 0.004 |
|  | HFD × NPY | 0.15 (1, 37) | 0.704 | -3.65, 5.34 | 0.004 |
| Food intake 24 h | HFD | 0.85 (1, 38) | 0.362 | -2.23, 0.83 | 0.021 |
|  | NPY | 0.56 (1, 38) | 0.458 | -0.96, 2.10 | 0.014 |
|  | HFD × NPY | 0.11 (1, 38) | 0.738 | -3.57, 2.55 | 0.003 |
| Food intake 48 h | HFD | 0.07 (1, 37) | 0.790 | -1.41, 1.08 | 0.002 |
|  | NPY | 0.56 (1, 37) | 0.460 | -0.78, 1.70 | 0.014 |
|  | HFD × NPY | 0.80 (1, 37) | 0.376 | -3.58, 1.39 | 0.021 |
| Body weight 24 h | HFD | 12.99 (1, 38) | <0.001 | -3.60, -1.01 | 0.248 |
|  | NPY | 0.11 (1, 38) | 0.741 | -1.51, 1.08 | 0.002 |
|  | HFD × NPY | 0.93 (1, 38) | 0.342 | -3.82, 1.36 | 0.018 |
| Body weight 48 h | HFD | 16.86 (1, 37) | <0.001 | -3.70, -1.26 | 0.308 |
|  | NPY | 0.01 (1, 37) | 0.931 | -1.17, 1.28 | 0.000 |
|  | HFD × NPY | 0.29 (1, 37) | 0.596 | -3.09, 1.80 | 0.005 |
| **Short-term (3 h) ingestive behaviour and body weight (Fig 3)** | | | | | |
| Sucrose intake [log_10_(Y)] | HFD | 0.10 (1, 40) | 0.753 | −0.18, 0.25 | 0.002 |
|  | NPY | 5.16 (1, 40) | 0.029 | 0.03, 0.46 | 0.102 |
|  | HFD × NPY | 5.51 (1, 40) | 0.024 | -0.94, -0.07 | 0.109 |
| Sucrose preference | HFD | 0.27 (1, 40) | 0.606 | −9.06, 15.34 | 0.005 |
|  | NPY | 2.89 (1, 40) | 0.097 | −1.93, 22.47 | 0.056 |
|  | HFD × NPY | 8.34 (1, 40) | 0.006 | -59.25, -10.45 | 0.162 |
| Food intake [log_10_(Y+1)] | HFD | 8.25 (1, 40) | 0.006 | −0.20, −0.04 | 0.156 |
|  | NPY | 4.13 (1, 40) | 0.049 | 0.0004, 0.17 | 0.078 |
|  | HFD × NPY | 0.90 (1, 40) | 0.348 | -0.25, 0.09 | 0.017 |
| Body weight | HFD | 10.09 (1, 40) | 0.003 | −0.44, −0.10 | 0.160 |
|  | NPY | 13.85 (1, 40) | <0.001 | 0.14, 0.49 | 0.219 |
|  | HFD × NPY | 0.12 (1, 40) | 0.729 | -0.40, 0.28 | 0.002 |
| **Hypothalamic markers and circulating corticosterone (Fig 4)** | | | | | |
| NPY peptide | HFD | 9.71 (1, 36) | 0.004 | 1.77, 8.36 | 0.209 |
|  | NPY | 0.70 (1, 36) | 0.410 | −1.94, 4.65 | 0.015 |
|  | HFD × NPY | 0.09 (1, 36) | 0.769 | -5.63, 7.55 | 0.002 |
| *Npy* | HFD | 29.28 (1, 36) | <0.001 | 0.25, 0.56 | 0.441 |
|  | NPY | 0.81 (1, 36) | 0.375 | −0.08, 0.22 | 0.012 |
|  | HFD × NPY | 0.27 (1, 36) | 0.603 | -0.22, 0.38 | 0.004 |
| *Il1b* | HFD | 3.69 (1, 37) | 0.062 | −0.47, 0.01 | 0.088 |
|  | NPY | 0.54 (1, 37) | 0.467 | −0.16, 0.33 | 0.013 |
|  | HFD × NPY | 0.58 (1, 37) | 0.451 | -0.67, 0.30 | 0.014 |
| *Nr3c1* | HFD | 1.95 (1, 38) | 0.170 | -0.20, 0.04 | 0.056 |
|  | NPY | 2.46 (1, 38) | 0.125 | −0.03, 0.21 | 0.044 |
|  | HFD × NPY | 1.71 (1, 38) | 0.200 | -0.08, 0.38 | 0.039 |
| *Crh* | HFD | 0.11 (1, 38) | 0.746 | −0.15, 0.21 | 0.003 |
|  | NPY | 0.16 (1, 38) | 0.695 | −0.14, 0.21 | 0.004 |
|  | HFD × NPY | 4.02 (1, 38) | 0.052 | -0.003, 0.71 | 0.095 |
| Corticosterone [log10(Y)] | HFD | 4.67 (1, 35) | 0.038 | 0.02, 0.52 | 0.098 |
|  | NPY | 2.89 (1, 35) | 0.098 | -0.47, 0.04 | 0.061 |
|  | HFD × NPY | 5.58 (1, 35) | 0.024 | -1.10, -0.08 | 0.118 |
| **Intranasal NPY in *Npy2r*-KO mice (Fig 5)** | | | | | |
| Sucrose intake | NPY | 0.02 (14) | 0.988 | −0.045, 0.046 | <0.001 |
| Sucrose preference | NPY | 0.96 (14) | 0.353 | −7.15, 18.78 | 0.062 |
| Food intake [log_10_(Y)] | NPY | 1.46 (17) | 0.163 | -0.59, 0.11 | 0.111 |
| Body weight [log_10_(0.2-Y)] | NPY | 1.02 (17) | 0.322 | -0.17, 0.49 | 0.058 |
| Corticosterone | NPY | 1.67 (14.82) Welch-corrected | 0.116 | −2.33, 19.04 | 0.158 |

HFD, high-fat diet; NPY, neuropeptide Y; KO, knockout; CI, confidence interval of the mean difference; η², eta squared; η²_p_, partial eta squared. *F* (df) and *p*-values from one-way or two-way ANOVA; transformations applied before analysis are indicated in parentheses. Gene symbols in italics denote hypothalamic mRNA expression. –, not reported.
